# Rhizarian ‘Novel Clade 10’ Revealed as Abundant and Diverse Planktonic and Terrestrial Flagellates, including *Aquavolon* n. gen.

**DOI:** 10.1111/jeu.12524

**Published:** 2018-05-14

**Authors:** David Bass, Denis Victorovich Tikhonenkov, Rachel Foster, Patricia Dyal, Jan Janouškovec, Patrick J. Keeling, Michelle Gardner, Sigrid Neuhauser, Hanna Hartikainen, Alexandre P. Mylnikov, Cédric Berney

**Affiliations:** ^1^ Department of Life Sciences The Natural History Museum Cromwell Road London SW7 5BD UK; ^2^ Centre for Environment, Fisheries and Aquaculture Science (Cefas) Barrack Road The Nothe Weymouth DT4 8UB UK; ^3^ Papanin Institute for Biology of Inland Waters Russian Academy of Sciences Borok 152742 Russia; ^4^ Botany Department University of British Columbia Vancouver BC V6T1Z4 Canada; ^5^ Department of Genetics, Evolution and Environment University College London Gower Street London WC1E 6BT UK; ^6^ Institute of Microbiology University of Innsbruck Technikerstraße 25 Innsbruck 6020 Austria; ^7^ Present address: Sorbonne Université & CNRS UMR 7144 (AD2M) Station Biologique de Roscoff Place Georges Teissier Roscoff 29680 France

**Keywords:** 18S rRNA, *Aquavolon dientrani*, *Aquavolon hoantrani*, Aquavolonida, Cercozoa, environmental sequencing, Rhizaria, *Tremula*

## Abstract

Rhizarian ‘Novel Clade 10’ (NC10) is frequently detected by 18S rRNA gene sequencing studies in freshwater planktonic samples. We describe a new genus and two species of eukaryovorous biflagellate protists, *Aquavolon hoantrani* n. gen. n. sp. and *A. dientrani* n. gen. n. sp., which represent the first morphologically characterized members of NC10, here named Aquavolonida ord. nov. The slightly metabolic cells possess naked heterodynamic flagella, whose kinetosomes lie at a right angle to each other and are connected by at least one fibril. Unlike their closest known relative *Tremula longifila*, they rotate around their longitudinal axis when swimming and only very rarely glide on surfaces. Screening of a wide range of environmental DNA extractions with lineage‐specific PCR primers reveals that Aquavolonida consists of a large radiation of protists, which are most diversified in freshwater planktonic habitats and as yet undetected in marine environments. Earlier‐branching lineages in Aquavolonida include less frequently detected organisms from soils and freshwater sediments. The 18S rRNA gene phylogeny suggests that Aquavolonida forms a common evolutionary lineage with tremulids and uncharacterized ‘Novel Clade 12’, which likely represents one of the deepest lineages in the Rhizaria, separate from Cercozoa (Filosa), Endomyxa, and Retaria.

SEQUENCING of the 18S rRNA gene from environmental samples has revealed a large diversity of undescribed protists in the vast majority of habitat types sampled (Richards and Bass 2005). Some of these are regularly detected and acquire their own quasi‐taxonomic status, e.g. the various MAST (marine stramenopiles; Massana et al. 2004) and MALV (marine alveolates; López García et al. 2001) lineages, and the LKM11 group related to fungi (Lara et al. 2010; Jones et al. 2011). Likewise, the first environmental sequencing survey of the phylum Cercozoa (Bass and Cavalier‐Smith 2004) revealed novel lineages that were too distantly related to known taxa to infer anything about their biology; they have since been referred to as cercozoan Novel Clades (NCs) and numbered 1 to 9.

Since 2004 several of these initial NCs have been identified by cell isolation or culturing efforts: the order Marimonadida (Howe et al. 2011) accounts for most of NC1, including genera *Pseudopirsonia* (Kühn et al. 2004), *Auranticordis* (Chantangsi et al. 2008), and *Abollifer* (Shiratori et al. 2014); NC2 has recently been shown to include the heterotrophic flagellate *Quadricilia* (Yabuki and Ishida 2018), the first member of NC4 was shown to be *Trachyrhizium* (Shiratori and Ishida 2016), which forms a clade with *Lecythium* and *Diaphoropodon* (Dumack et al. 2018); NC6 belongs to a group including *Metopion*,* Metromonas*, and *Micrometromonas* (Howe et al. 2011; this study); NC7 includes Pansomonadida (*Aurigamonas* and *Agitata*; Bass et al. 2009a); and NC8 is part of Vampyrellida (Bass et al. 2009b; Hess et al. 2012; Berney et al. 2013). NCs 3 and 5 remain uncharacterized, although Bass et al. (2009b) showed that NC5 is a subclade of Granofilosea, a class of filose amoebae. More data availability and improved phylogenetic methods have also shown that ‘Basal Group T’ in Bass and Cavalier‐Smith (2004) is more morphologically and phylogenetically diverse than that label suggests, including the flagellate *Discomonas* (Chantangsi and Leander 2010) and the testate amoeba *Penardeugenia* (Dumack et al. 2017).

Further NCs were defined in Bass et al. (2009b), the most relevant here being NCs 10, 11, and 12. These lineages sometimes group together with moderate support. Their exact phylogenetic position within Rhizaria remains unresolved, but they typically appear distinct from both core Cercozoa (previously Filosa) and Endomyxa (Bass et al. 2009b; Howe et al. 2011). On the basis of recent phylogenomic analyses placing the root of the rhizarian radiation between Cercozoa (Filosa) and Endomyxa + Retaria (Sierra et al. 2016; Krabberød et al. 2017), this group may therefore form one of the deepest branching rhizarian lineages. NC11 was shown by Howe et al. (2011) to contain *Tremula longifila*, a biflagellate gliding cell (ATCC 50530), which along with its direct relatives inhabits freshwater sediments and soil. Cellular information for NC12 is still lacking, despite its frequency and diversity in a wide range of environments. NC12 was originally defined on the basis of sequences analyzed in Bass et al. 2009b. Since then a larger diversity of related environmental sequences has been revealed, analysis of which suggests the original NC12 may correspond to two separate, strongly supported clades plus some unaffiliated, single‐sequence lineages.

Likewise, NC10 has so far remained uncharacterized. It is, however, remarkable for being very frequently and almost exclusively detected in freshwater planktonic samples, from the Antarctic to subtropical lakes. Table S1 provides a list of all NC10 environmental clones we identified in GenBank at the onset of this work, with information about their provenance. Key examples of these are as follows: LG21‐01 and LG01‐12 in Richards et al. (2005), A50 in Lefranc et al. (2005) (not A51, which is a vampyrellid; Berney et al. 2013), ‘Novel clade I’ in Lefèvre et al. (2007, 2008) (except PCC4AU2004, which belongs to NC12), all of the cercozoan sequences in Fig. 4 of Lepère et al. (2008) (except *Cercomonas* and PCC4AU2004), the ‘Cercozoa freshwater clade’ in Taib et al. (2013) (except again PCC4AU2004), the OTUs identified as belonging to NC10 in Simon et al. (2015) and Yi et al. (2017), and in Lara et al. (2015), in which NC10 lineages are shown to be well but variably represented in peat bogs in Argentinian Tierra del Fuego.

In this study we investigated the environmental diversity and ecology of NC10 by using clade‐specific PCR primers to screen freshwater, soil, and marine environmental DNA samples, as well as fluorescent in situ hybridization (FISH). Furthermore, phylogenetic analysis of 18S rRNA gene sequences of flagellates isolated from a reservoir and wetland in Vietnam showed them to belong to NC10. These were described using light and transmission electron microscopy (TEM) as two new species within the new genus *Aquavolon*. In the light of 18S rRNA gene sequence signatures shared by all NC10 lineages presented in this study, morphological observations on our isolates, and the habitat and likely phenotype of other NC10 members, we formally rename the group Aquavolonida, ord. nov.

## Materials and Methods

### Environmental DNA screening and NC10 clone library sequencing

DNA samples from filtered lake water from the English Lake District (< 2 μm and 2–20 μm size fractions), river biofilms, and soils that were used for the study of Grossmann et al. (2016) were also used for this study: DNA was extracted from filtered water using the Qiagen Blood & Tissue DNA Extraction Kit (Qiagen, Hilden, Germany). DNA was extracted from biofilms newly formed on ceramic tiles placed in the River Lambourn, UK by a standard CTAB/phenol‐chloroform extraction method, and from soils using the FastDNA SPIN Kit for Soil (MP Biomedicals LLC, UK). Marine environmental DNA extractions previously used in other studies (Bass and Cavalier‐Smith 2004; Bass et al. 2009b; Berney et al. 2013; Hartikainen et al. 2014a,b; Logares et al. 2014) were also used for screening.

Two PCR strategies with distinct levels of specificity to NC10 were used for environmental DNA screening, both based on a two‐step, nested PCR approach using distinct sets of PCR primers (see Results for the rationale and specificity of these PCR strategies). Strategy I: first PCR with primers C7f‐NC10 and sB2n, nested PCR with primers V8f‐NC10 and EndoR1. Strategy II: first PCR with primers V2f‐NC and C9r‐NC, nested PCR with primers C5f‐NC and V8r‐NC. Construction of Endomyxa‐enriched clone libraries was achieved using a two‐step, semi‐nested PCR approach: first PCR with primers s1259F and sB2n, semi‐nested PCR with primers s1259F and EndoR1. PCR primers corresponding to the NC10‐specific FISH probes we designed were used to verify their strict specificity to a subset of NC10 using the following semi‐nested PCR approach: first PCR with primers V4f‐NC10 and C7r‐NC10, semi‐nested PCR with primers V4f‐NC10 and C6r‐NC10. Table S2 provides the sequence and position of these primers.

PCR amplifications were done in a total volume of 25 μl with an amplification profile typically consisting of 2 min at 95 °C, followed by 30 cycles (first PCR) or 25 cycles (nested PCR) of 30 s at 95 °C, 30 s at 65 °C, and 2 min at 72 °C, followed by 5 min at 72 °C for the final extension. Purified amplicons of the appropriate length were cloned into TOPO‐TA vectors and screened using primers M13for (5′‐CGT TGT AAA ACG ACG GCC AGT‐3′) and M13rev (5′‐CAC AGG AAA CAG CTA TGA CCA‐3′). Positive PCR products were cleaned using a polyethylene glycol (PEG) protocol and sequenced using the same primers on an ABI‐377 sequencer. Sequences were edited in FinchTV (http://www.geospiza.com/finchtv/) and aligned in BioEdit (Hall 1999).

### Isolation of protist strains

Colp‐16, Kat‐1, and Colp‐11 were isolated as single cells by a glass micropipette. Cells were picked from Petri dishes and washed in a series of 20 μm droplets of Pratt's medium. Clone Colp‐16 was isolated from a near‐shore water sample with bottom detritus from Suoi Da Reservoir (Hồ Suối Đá, viet.), Bình Thuận Province, S.R. Vietnam (11.122526N, 108.183158E) collected on May 10th 2013 (Water Temp. 33.08 °C, pH 8.42, conductivity 96 μS/cm, TDS 48 ppm, DO 6.30 ppm). Clone Kat‐1 was isolated from a water sample containing decaying plant material from a wetland near farmland (11.509471N, 107.365586E), Đắc Lua Village, Dong Nai Province, S.R. Vietnam collected on May 15th 2012 (Water Temp. 27.5 °C, pH 5.77, TDS 36 ppm, DO 1.4 ppm). Clone Colp‐11 was isolated from a water sample with bottom detritus in a small boggy pond surrounded by pine trees within white sand dunes (11.066331N, 108.426602E) near Bau Trang lake, Bình Thuận Province, S.R. Vietnam. Sample was collected on May 9th 2013 (Water Temp. 36.72 °C, pH 6.17, conductivity 30 μS/cm, TDS 15 ppm, DO 4.36 ppm). Hydrochemistry characteristics were measured using the multiparameter meter HI 9828 (Hanna instruments). Field studies in Vietnam were conducted under permits issued by the administration of Cát Tiên National Park, Vietnam, and authorized by Vietnam‐Russian Tropical Centre, Coastal Branch (Nha Trang, Vietnam).

### Culturing and microscopy

All strains were propagated on the bodonid *Parabodo caudatus* strain BAS‐1 or the chrysophyte *Spumella* sp. strain OF‐40 (both freshwater) grown in Pratt's medium by using the bacterium *Pseudomonas fluorescens* as food (Tikhonenkov et al. 2014). Cells grown in laboratory cultures were harvested following peak abundance after eating most of the prey and collected by centrifugation (10,000 *g* for 10 min, room temperature). Genomic DNA was extracted from fresh cells using the Epicentre DNA extraction kit (Cat. No. MC85200). All three cultures died after 1 year of continuous passaging.

Light microscopy observations of Colp‐16 were made by using a Zeiss AxioScope A.1 equipped with a DIC and phase contrast water immersion objectives (63×) and the analog video camera AVT HORN MC‐1009/S. Light microscopy observations of Kat‐1 and Colp‐11 were made by using a Zeiss AxioPlan2 Imaging microscope equipped with a DIC contrast objective (40×) and the video camcorder Canon XL H1s.

For transmission electron microscopy (TEM), cells were centrifuged, fixed in 0.6% glutaraldehyde and 2% OsO_4_ (final concentration) prepared using a 0.1 M cacodylate buffer (pH 7.2) at 1 °C for 30–60 min, and dehydrated in an alcohol and acetone series (30, 50, 70, 96, and 100%; 20 min in each step). Finally, cells were embedded in a mixture of araldite and epon (Luft 1961). Ultrathin sections were obtained using a LKB ultramicrotome. TEM observations were done by using the JEM‐1011 (Japan) electron microscope.

### Full 18S rRNA gene sequencing and phylogenetic analyses

The full 18S rRNA gene sequence of isolates Colp‐16 and Kat‐1 was amplified using general eukaryotic primers 18SFU and 18SRU (Tikhonenkov et al. 2016), while that of isolate Colp‐11 was obtained with general eukaryotic primers PF1 and FAD4 (Tikhonenkov et al. 2014). Products were then cloned and sequenced by Sanger dideoxy sequencing. The first half of the 18S rRNA gene of environmental clone Wsoil (one of the NC10 phylotypes identified in Endomyxa‐enriched clone libraries from soil samples) was amplified from the corresponding soil DNA extraction and sequenced using an Endomyxa‐biased forward primer sA4‐en2 (5′‐CWG TGA AAC TGC RGA TGG‐3′) and a phylotype‐specific reverse primer in hypervariable region V7, V7r‐nc14 (5′‐CCT CTA AGA AGT CAT TCT CCA ACG‐3′). The 18S rRNA gene sequences generated in this study were deposited in the NCBI GenBank database under accession numbers MG760572, MG818163‐MG818165, and MG818835‐MG818856.

Sequence alignments including our new cloned and isolate‐derived sequences and previously published sequences representative of the known diversity of Cercozoa and Endomyxa were generated using MAFFT v. 7 (e‐ins‐i algorithm) (Katoh and Standley 2013) and masked to omit ambiguously aligned positions. Phylogenetic analyses were performed on the CIPRES Science Gateway (Miller et al. 2010). Maximum likelihood analyses were performed with the program RaxML v 8 (Stamatakis et al. 2008; Stamatakis 2014) and Bayesian analyses using MrBayes v 3.2.5 (Ronquist et al. 2012). Two separate MC^3^ runs with randomly generated starting trees were carried out for 5 million generations each with one cold and three heated chains. The evolutionary model applied included a GTR substitution matrix, a four‐category autocorrelated gamma correction, and the covarion model. All parameters were estimated from the data. Trees were sampled every 1,000 generations. The first 1.25 M generations were discarded as burn‐in (trees sampled before the likelihood plots reached stationarity) and a consensus tree was constructed from the remaining sample.

### TSA‐FISH and flagellar staining

Newly collected freshwater plankton samples from the English Lake District in which NC10 had been frequently detected by PCR were prepared for TSA‐FISH and flagella antibody staining. Two‐hundred milliliter samples were fixed with formaldehyde and filtered through 0.6 μm pore‐size polycarbonate filters. The filters were stored at −80 °C in the dark until being processed. Prior to hybridization the filters were sliced into small wedges and equilibrated in 50 μl PBS and 0.3% triton in a humid chamber for 1 h at 40 °C. Then to minimize endogenous peroxidases the filters were incubated for 10 mins with 10 μl of 3% H_2_O_2_. The filters were washed three times with fresh wash buffer (0.9 M NaCl, 5 mM EDTA, 20 mM Tris‐HCl (pH 7.5), and 0.01% sodium dodecyl sulfate).

Hybridization was carried out by covering filter pieces with 20 μl of hybridization buffer (35% deionized formamide, 0.9 M NaCl, 20 mM Tris‐HCl (pH 7.5), 0.01% sodium dodecyl sulfate, and 10% blocking reagent) containing 2 μl of horseradish peroxidase (HRP)‐labeled probe (stock at 50 ng/μl) and incubating in a humid chamber at 40 °C overnight. Two oligonucleotide probes specific to the planktonic clade of NC10 were designed: NC10‐V4 (5′‐GTATTCACAGCAATAAGCCTGCTT‐3′) and more specific NC10‐C6 (5′‐CTCCACTTCTTGGGTGCC‐3′). The probe EUK516 (5′‐ACCAGACTTGCCCTCC‐3′) (Amann et al. 1990) was used as a positive control for each batch of hybridizations. To test for any false positives caused by endogenous peroxidases further procedures were undertaken: (i) a control without probe (no fluorescence was detected) and (ii) incubation of filters with 3% hydrogen peroxide to remove peroxidases. The formamide concentration was also reduced in test hybridizations to determine whether the patchy‐specific probe signal was caused by too stringent hybridization conditions.

After hybridization filters had three 5‐min wash steps with wash buffer (as before) at 42 °C. Filters were equilibrated in TNT buffer (0.1 M Tris‐HCl (pH 7.5), 0.15 M NaCl, 0.05% Tween 20) for 15 min at room temperature. TSA Fluorescein was made up as per kit instructions (Perkin Elmer) and 50 μl of fluorophore working solution was added to each filter and incubated for 30 min at room temperature in the dark. The filters were then washed in prewarmed TNT buffer and incubated for 15 min at 55 °C replacing with fresh buffer every 5 min. The filters were removed and air dried (still in the dark).

Immediately after the TSA‐FISH protocol the filters were fixed in 4% formaldyde in PBS for 10 min. Samples were rinsed in PBS and incubated in blocking solution (1× PBS with 4% BSA, 0.3% Triton X, and 0.02% sodium azide) for 1 h at 4 °C. Primary antibody, monocolonal antiacylated tubulin antibody produced in mouse (Sigma) was diluted 1:200 with blocking solution and incubated for 3 h at 4 °C. Samples were washed five times with PBS and 0.3% triton X. Secondary antibody goat antimouse igG TRITC (abcam) was diluted 1:400 in blocking solution and sample was incubated for 1 h at 4 °C. The filters were washed five times in PBS and 0.3% triton X and air dried on slides for 5 min. They were then mounted with Vectasheld containing DAPI and the coverslips sealed, and examined under a Nikon A1 confocal microscope.

## Results

### Diversity and habitat range of Aquavolonida (=NC10)

We set out to explore the diversity and ecology of Aquavolonida by screening environmental DNA extractions using two different PCR strategies (Tables 1 and S2). First, we designed PCR primers targeting ‘core’ Aquavolonida, i.e. the diversity of NC10 environmental clones that was publicly available at the onset of this study (PCR strategy I; see Table S1). Applying PCR strategy I to a range of terrestrial and marine samples showed that Aquavolonida could only be identified in freshwater habitats, although not exclusively in plankton samples: we also detected Aquavolonida phylotypes in freshwater sediments and in association with river biofilms (Table 1). However, the PCR primers used in PCR strategy I could have so high a specificity to previously known, ‘core’ Aquavolonida sequences that they may not allow detection of more divergent Aquavolonida lineages, if any.

**Table 1:** Detection of Aquavolonida 18S rRNA phylotypes in various habitat types using two different PCR strategies. Dashes indicate that the primer strategy did not amplify Aquavolonida from that habitat type

Habitat type	PCR strategy I – ‘core’ Aquavolonida	PCR strategy II – general Aquavolonida diversity	
	Positive samples	Positive samples	Aquavolonida lineage(s) detected
Marine sediment	0 of 32	0 of 32	–
Marine plankton	0 of 32	0 of 32	–
Estuarine gradient	1 of 16^a^	0 of 16	–
Soils	0 of 16^b^	11 of 16	NC10‐D (94% of clones) and NC10‐B (6% of clones)
Freshwater sediment	8 of 16	14 of 16	NC10‐A (63% of clones) and NC10‐B (37% of clones)
Freshwater river biofilm	7 of 8	7 of 8	NC10‐A (80% of clones) and NC10‐B (20% of clones)
Freshwater plankton	16 of 16	16 of 16	NC10‐A (67% of clones) and NC10‐B (33% of clones)

a The only positive sample was from the freshwater end of the estuarine gradient.

b On soil samples this PCR strategy can generate false positives (amplification of lobose amoebae with the primers used in the first PCR).

To address this issue, we relied on the results of preliminary surveys of general endomyxan diversity in various environments that were conducted at the NHM as part of a research project on Endomyxa (data not shown). This screening effort was designed to explore as widely as possible the diversity of Endomyxa and NCs 10 to 12 (using a broad Cercozoa+Endomyxa‐specific forward primer), while excluding the overwhelming diversity of core Cercozoa (Filosa) using ‘anti‐Filosa’ reverse primers (see Table S2). In clone libraries generated with these primers from soil samples, we identified (at low frequency compared to other soil endomyxans like Vampyrellida) two very distinct, novel phylotypes (clones Dsoil and Wsoil) that showed specific phylogenetic affinities to ‘core’ Aquavolonida. We designed a Wsoil‐specific reverse primer to obtain its full 18S rRNA gene sequence from the corresponding soil DNA extraction. In parallel, preliminary phylogenetic analysis of the full 18S rRNA gene sequences of flagellates isolated from a reservoir and wetland in Vietnam (morphologically described below) showed them to also be related to ‘core’ Aquavolonida, some of them with sequences highly similar to clone Dsoil.

**Figure 1:**
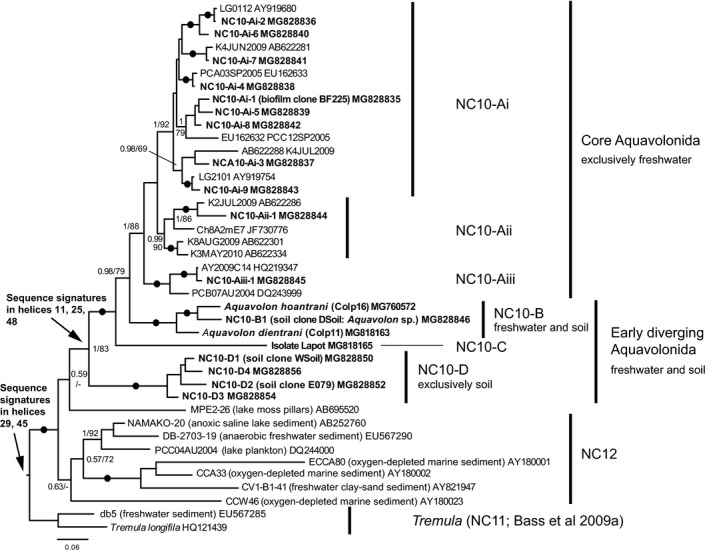
Bayesian SSU rRNA gene phylogeny of Aquavolonida (NC10) diversity, including new taxa *Aquavolon hoantrani* and *A. dientrani*, with tremulids and NC12 used as outgroups. Bayesian Posterior Probabilities (BPP) and Maximum Likelihood (ML) bootstrap values are indicated on nodes; black filled circles indicate values of BPP ≥ 0.95 and ML bootstrap ≥ 95%. Sequences generated in this study are shown in bold text.

All of these relatives to ‘core’ Aquavolonida would be missed by the primers used in PCR strategy I. We therefore designed PCR strategy II to target more broadly the diversity of Aquavolonida, and applied these primers to the same habitat types tested with PCR strategy I (Table 1). Figure 1 shows a Bayesian phylogenetic tree of the diversity of Aquavolonida 18S rRNA gene phylotypes identified with PCR strategy II, using tremulids and NC12 as outgroups. ‘Core’ Aquavolonida, which comprises all previously identified sequences from GenBank, all our cloned sequences from freshwater planktonic samples, and a few cloned sequences from freshwater sediments and river biofilm, form the bulk of Aquavolonida diversity and are here labeled lineage Aquavolonida NC10‐A. The NC10‐A radiation is subdivided into three phylogenetically distinct subgroups Ai to Aiii; Ai is the most diverse both among GenBank sequences and our clone libraries. No morphological information is available for any of them; however, Ai is the subgroup targeted by our FISH probes (see below).

Below ‘core’ Aquavolonida, we identified a grade of three earlier‐branching lineages (B‐D) that by contrast were not identified in freshwater planktonic samples. Aquavolonida NC10‐B comprises most of our isolated strains, and the two new species described in this study (see below). This lineage was detected in soils, freshwater sediments, and river biofilm. The single, divergent sequence in Aquavolonida NC10‐C is another isolated strain, a gliding flagellate with a similar gross morphology and feeding mode to the new species, to be formally described elsewhere. The earliest branching lineage (Aquavolonida NC10‐D) is composed entirely of environmental sequences (including clone Wsoil), and has so far exclusively been detected in soil samples. Even though PCR strategy II targets a much broader diversity of organisms than PCR strategy I, it did not detect any lineage in addition to those that were used to design the primers.

### Morphology of the novel isolates

The three novel isolates Colp‐16, Kat‐1, and Colp‐11 from freshwater biotopes of Southern Vietnam all belong to lineage Aquavolonida NC10‐B. At present this is the only Aquavolonida lineage for which we have data on both cell morphology and habitat range. We create the new genus *Aquavolon* (see the Taxonomic Summary below) to accommodate this lineage detected in soils, freshwater sediments, and river biofilm samples. Isolate Colp‐16 is distinct from the other two isolates both morphologically and in its 18S rRNA gene sequence and is here described as *Aquavolon hoantrani* n. gen. n. sp. (type species of the genus). The other two isolates are morphologically undistinguishable and have identical 18S rRNA gene sequences; Kat‐1 is described as *Aquavolon dientrani* n. sp. and Colp‐11 is regarded as a co‐specific isolate.

### *Aquavolon hoantrani* n. gen. n. sp. (clone Colp‐16)

#### Light microscopy

**Figure 2:**
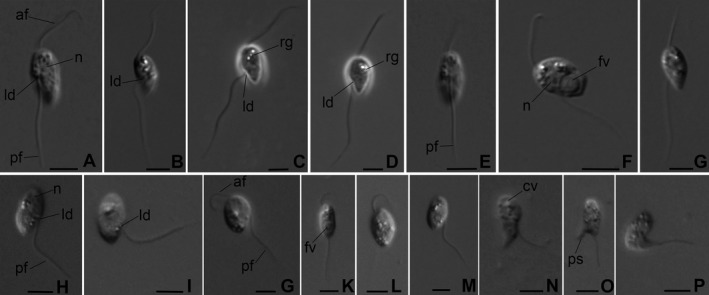
External morphology of *Aquavolon hoantrani* n. gen., n. sp. isolate Colp‐16 (**A‐G**) and *A. dientrani* n. sp. isolate Kat‐1 (**H‐P**). af, anterior flagellum; cv, contractile vacuole; fv, food vacuole; ld, lateral depression; n, nucleus; pf, posterior flagellum; ps, pseudopodium; rg, light‐refracting granules. Scale bar: 5 μm for all figures.

Cells are elongated‐oval, slightly metabolic, not flattened, 7–10 μm long, and 3.5–5 μm wide. The anterior end of the cell is usually wider than the posterior one and both are roundish. No anterior rostrum. A remarkable lateral depression is situated in the middle lateral point of the cell body (Fig. 2A‐D). Pseudopodia were not observed.

Two long heterodynamic flagella originate near the anterior cell end (Fig. 2E). The anterior flagellum is 1.5–2 times longer than its cell body length, makes weak waving movements, and directs forward during cell swimming. The posterior flagellum is about 2–3 times the cell length, lies along the cell body at the anterior part of the cell, stands apart from the cell at the level of lateral depression, directs backward, and trails behind the cell (Fig. 2B‐D). A contractile vacuole (not shown) and nucleus (Fig. 2A,F) are located subapically. The cytoplasm contains light‐refracting granules (Fig. 2C,D). Cells usually swim rapidly near the bottom of Petri dishes and very rarely glide on the surface. The anterior flagellum does not wrap around the anterior part of the body.

*Aquavolon hoantrani* is a predator, feeding on other flagellates (e.g. bodonids) and perished in the absence of eukaryotic prey. However, *A. hoantrani* did not feed very actively and never captured all available prey cells. Cannibalism was not observed. A large food vacuole is formed at the posterior end of cell body following the feeding and cells become wider (Fig. 2F). Division was not observed. Reproduction or resting cysts were not seen in the culture.

#### Ultrastructure

**Figure 3:**
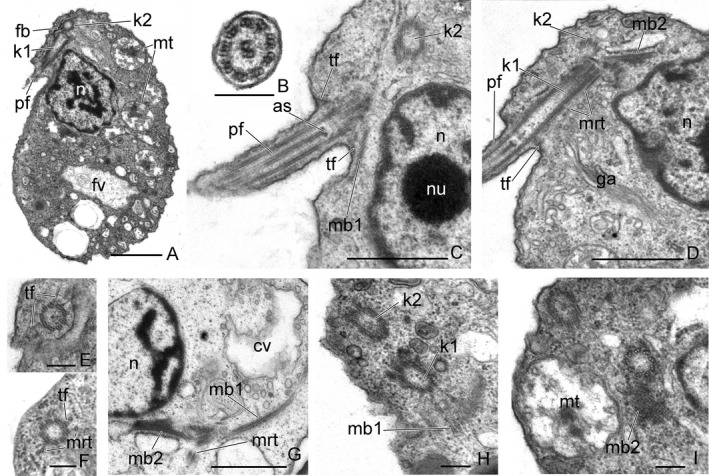
The general view, anterior end of the cell, and some flagellar root structures of *Aquavolon hoantrani*. **A.** The longitudinal section of the cell. B. Cross‐section of the flagellum. **C‐D.** Longitudinal sections of the anterior end of the cell. **E‐H.** Relative arrangement of the kinetosomes. **I.** Some microtubular bands near the nucleus. as, axosome; cv, contractile vacuole; fv, food vacuole; ga, Golgi apparatus; k1, kinetosome 1; k2, kinetosome 2; mb1, microtubular band 1; mb2, microtubular band 2; mt, mitochondria; mrt, microtubules; n, nucleus; nu, nucleolus; pf, posterior flagellum; tf, transition fibers. Scale bar: (A, C, D, I) 1 μm, (B, E – H) 0.2 μm.

The cell surface is covered only with a plasma membrane. Two smooth heterodynamic flagella with typical axonemes (9 + 2) insert subapically (Fig. 3A‐D). The flagella basal bodies lie approximately at a right angle to each other and are connected by at least one fibril (Fig. 3A). Two central microtubules contact with the axosome (Fig. 3C). Kinetosome 1 (basal body of posterior flagellum) is very long (more than 1 μm) (Fig. 3D). The distal end of each kinetosome is connected with nine transition fibers to the plasma membrane (Fig. 3C‐F). Some microtubules (mrt) extend from the kinetosome to the plasma membrane (Fig. 3D, F, G). The cell possesses two wide microtubular bands. Microtubular band 1 begins from kinetosome 1 and contains up to 14 microtubules (Fig. 3C,G, H). Microtubular band 2 starts from kinetosome 2 and consists of 5–7 microtubules (Fig. 3D,G,I). The microtubules of this band are surrounded by dark amorphous material. The details of the arrangement of Microtubular bands have not been established.

**Figure 4:**
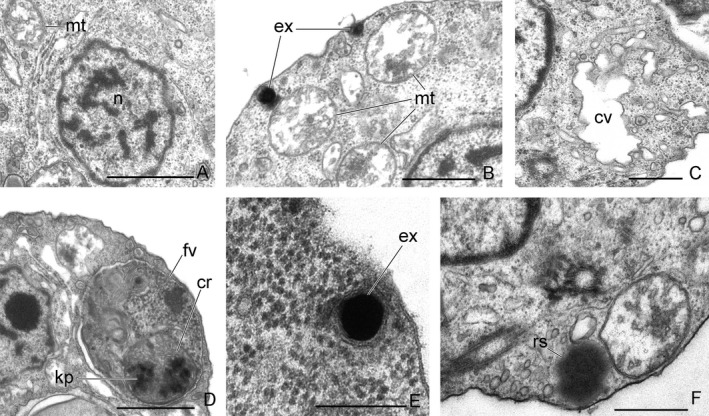
The nucleus and other cell structures of *Aquavolon hoantrani*. **A.** Nucleus. **B.** Mitochondria. **C.** Contractile vacuole. **D.** Food vacuole. **E.** Extrusome. **F.** Reserve substance. cr = mitochondrial cristae of *Parabodo caudatus*, cv, contractile vacuole; ex, extrusome; fv, food vacuole; kp, putative kinetoplast of *Parabodo caudatus;* mt, mitochondrion; n, nucleus; rs, reserve substance. Scale bar: (A) 1 μm, (B‐D, F) 0.5 μm, (E) 0.2 μm.

A nucleus with nucleolus is situated close to the kinetosomes (Fig. 3A, C, D, G, 4A). The narrowed end of the nucleus directs to the kinetosomes (Fig. 3A). Golgi apparatus of typical structure is located near the nucleus (Fig. 3D). Several mitochondria were observed to have tubular cristae (Fig. 3A, 4A, B). A contractile vacuole, surrounded by small vesicules, lies close to the nucleus (Fig. 3G, 4C). Food vacuoles contain the engulfed eukaryote prey at different stages of digestion. The remnants of *Parabodo caudatus* (see flat mitochondrial cristae and putative kinetoplast) are visible inside the food vacuole (Fig. 4D). *A. hoantrani* phagocytoses the cells of eukaryotic prey intact. The cytostome and cytopharynx or longitudinal groove for engulfment of prey are absent. Small spherical osmiophilic dark‐stained granules are embedded inside the vesicles (Fig. 4B, E). These structures likely represent extrusive organelles, which resemble muciferous bodies of some cercomonads and Pseudosporida. Spherical granules of a storage compound were found in the cytoplasm (Fig. 4F).

### *Aquavolon dientrani* n. sp. (Fig. 2H‐P) (clones Kat‐1 and Colp‐11)

The general morphology, body plan, and feeding are the same as in *A. hoantrani*. *A. dientrani* is also characterized by elongated‐oval, slightly metabolic, not flattened cells with wider anterior end and a lateral depression (Fig. 2H, I). Cells are slightly larger than in the other species and reach 8.5–11 μm. In contrast with *A. hoantrani*, the anterior flagellum of *A. dientrani* is very short, about the half of cell length or slightly longer (Fig. 2G‐L), making very fast flapping movements during swimming, and is often invisible. Sometimes the anterior flagellum wraps around the anterior part of the cell. The posterior flagellum is 2–2.5 times longer than the cell body, also stands apart from the cell at the level of the lateral depression and trails behind the cell.

A contractile vacuole and nucleus are situated close to the anterior end (Fig. 2H,N). Light‐refracting granules were observed in the cytoplasm. Cells usually swim with rotation around their axes, only rarely gliding on the substrate (Video S1). Sometimes after contact with the substrate, the cells can form short, relatively wide pseudopodia posteriorly (Fig. 2N, O), or produce a single ‘tail’ pseudopodium (Fig. 2P). Like *A. hoantrani*,* A. dientrani* captures other flagellates and perishes in the absence of eukaryotic prey. Division was not observed. Reproduction or resting cysts were not seen in the culture.

### TSA‐FISH of freshwater planktonic samples

**Figure 5:**
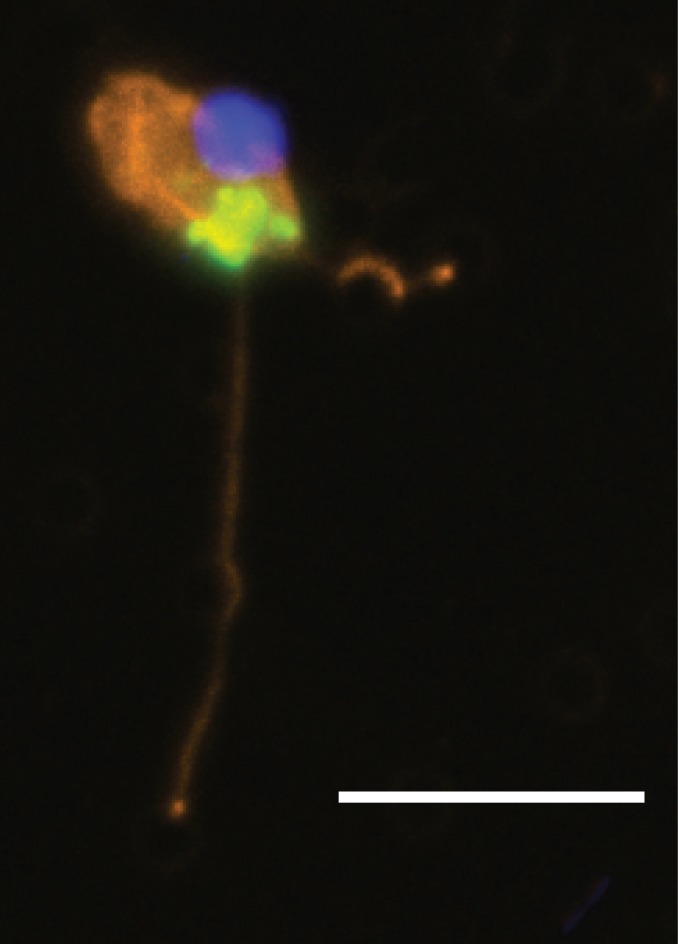
TSA‐FISH of putative lineage NC10‐Ai (‘core’) Aquavolonida cells from freshwater plankton (Derwent Water, Cumbria, UK). Nucleus = blue (DAPI), NC10‐specific TSA‐FISH signal = green, flagellar stain = orange. Confocal image focused in the plane of the green signal. Scale bar = 10 μm.

Two TSA‐FISH probes designed to target specific motifs in the variable region V4 and in the conserved region between V5 and V6 of the 18S rRNA (Table S2) were used to determine the morphology of the planktonic ‘core’ Aquavolonida (lineage NC10‐Ai; Fig. 1). Cells showing the green fluorophore of the specific probe were very infrequently seen on all filters examined. In contrast, the broadly targeted eukaryotic probe illuminated many cells on the same filters. Both Aquavolonida‐specific probes independently generated the same signal, i.e. flagellated cells of the same morphological type and within the size range given for the two *Aquavolon* spp., above. Unusually (and in contrast to the pan‐eukaryotic control), the ribosomal probe was in all cases localized to distinct areas in the posterior half of the cell (Fig. 5). Possible causes for this were tested in various ways (see Methods). Endogenous peroxidases were limited by hydrogen peroxide treatment, and tested for in a control hybridization without the probe; neither of these suggested that the localised signal was a false positive caused by the presence of peroxidases. Furthermore, the localised signal was consistent across the two independent probes tested and a range of formamide concentrations moderating the hybridization stringency.

### *Improved phylogeny of Aquavolonida* ord. nov

**Figure 6:**
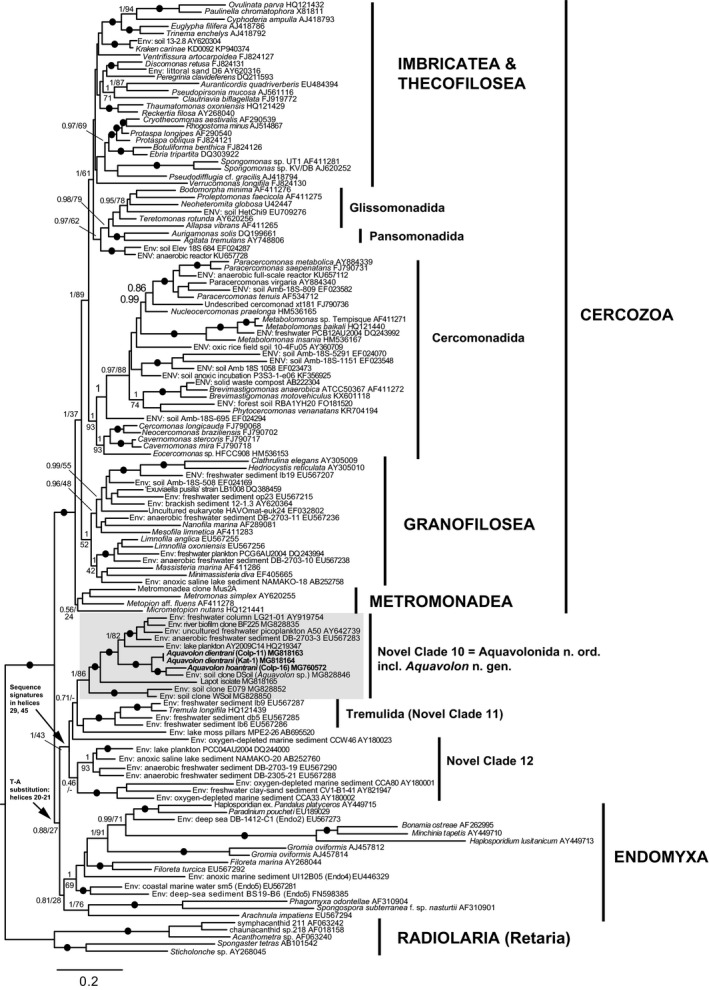
Bayesian SSU rRNA gene phylogeny showing the position of Aquavolonida, including new taxa *Aquavolon hoantrani* and *A. dientrani* (bold text), in relation to a broad selection of other rhizarians, including Cercozoa (=Filosa), Tremulida (=NC11), NC12, and Endomyxa, arbitrarily rooted on Radiolaria (shorter‐branched Retaria). Bayesian Posterior Probabilities (BPP) and Maximum Likelihood (ML) bootstrap values are indicated on nodes; black filled circles indicate values of BPP ≥ 0.95 and ML bootstrap ≥ 95%.

Using the new full‐length 18S rRNA gene sequences generated in this study, we reassessed the phylogenetic position of Aquavolonida within Rhizaria. Figure 6 shows a Bayesian phylogenetic analysis in which a strongly supported NC10 (Aquavolonida) forms a clade with Tremulida, the two main lineages comprising NC12, and two single‐sequence lineages from moss pillars in an Antarctic freshwater lake (clone MPE2‐26; Nakai et al. 2012) and an oxygen‐depleted marine environment (clone CCW46; Stoeck and Epstein 2003). This whole clade is sister to Endomyxa, albeit without strong support, but is clearly distinct from the rest of Cercozoa (=Filosa). Relationships within Endomyxa and Filosa are consistent with previous studies (e.g. Bass et al. 2009b).

Sequence signatures in the 18S rRNA gene (Table 2) clearly support the monophyly of Aquavolonida as defined here, i.e. comprising four main lineages NC10‐A (=‘core’ Aquavolonida), NC10‐B (=*Aquavolon* n. gen.), NC10‐C (=isolate Lapot), and NC10‐D (environmental lineage so far exclusively found in soils). These signatures are detailed below following the helix numbering in Wuyts et al. (2000) and were reflected in the primers we designed for PCR strategy II (see Tables 1 and S2). They consist of two complementary substitutions from U‐A to G‐C and from G‐C to C(U)‐G(A) in helix 11 (variable region V2, primer V2f‐NC), one complementary substitution from A‐T to T(C)‐G(A) in helix 48 (conserved region between V8 and V9, primer C9r‐NC), and a specific motif of two adjacent substitutions (AU instead of YG) in a nonbinding part of the 3′ stem of helix 25 (conserved region between V4 and V5, primer C5f‐NC). Some of these signatures can be found in part in other eukaryotic lineages, but their combination is uniquely found only in, and shared by, all NC10 lineages. Closely related groups (tremulids, NC12, and environmental clones MPE2‐26 and CCW46) all lack these signatures. The clade consisting of NC10, NC12, Tremulida, and environmental clones MPE2‐26 and CCW46 is supported by a complementary substitution of A‐U to C‐G in helix 29 (variable region V5) and a complementary substitution of A‐U to U‐A in helix 45 (variable region V8, primer V8r‐NC). Finally, a unique substitution from U to A in the loop between helices 20 and 21 characteristic of all Endomyxa clades (e.g. Vampyrellida, Phytomyxea, *Filoreta*,* Gromia*, and Ascetosporea) is also found in the clade comprising NC10, NC12, and Tremulida, supporting a closer relationship to Endomyxa than core Cercozoa (=Filosa).

**Table 2:** Sequence signatures observed in the 18S rRNA gene supporting the monophyly of Aquavolonida ord. nov. and its relationship with Tremulida and NC12

Helix and positions^a^	Specific sequence motif^b^	Consensus sequence in other eukaryotes
Helix 11, 229‐234 and 248‐252	[R(A)**G**C**Y**Y]–[R**R**G**C**^Y]	[R(A)UCGC]–[GCGA^Y]
Between helices 20 & 21, 537‐545	GCGGUAAUA	GCGGUAAUU
Helix 25, 866‐878 and 885‐900	[UCHDNGG(CCA)GAG]–[UUC(U**AU**GAU)UCNBKGA]	[UYMRYKG(YCA)GAG]–[UUC(UYGGAU)YYRYKRA]
Helix 29, 1016‐1028 and 1041‐1053	[**C**GR(GA)UBRGYSVW]–[UBBRYYDG(CA)YC**G**]	[AGG(GA)UYRGNRRR]–[YYYNYYRG(CA)CCU]
Helix 45, 1451‐1456 and 1506‐1511	[G**U**UGCD]–[BGCA**A**C]	[GAYRVR]–[YBYRUC]
Helix 48, 1565‐1573 and 1579‐1587	[AA(C)GCR**Y**KU]–[GM**R**YGC(A)UU]	[AR(C)GCRAGU]–[RCUYGC(R)YU]

a Helix numbering according to Wuyts et al. (2000), and positions given using NC10 environmental clone AY2009C14 (accession number HQ219347) as a reference.

b Bold and underlined: three complementary and two single‐sequence signatures that together are uniquely found in Aquavolonida ord. nov. and are used as a diagnostic feature of this order; bold: two complementary sequence signatures supporting a clade comprising Aquavolonida, Tremulida, and NC12; underlined: single‐sequence signature shared by this clade and Endomyxa, but not core Cercozoa (=Filosa).

## Discussion

At the onset of this study, NC10 (Bass et al. 2009a) was only known from environmental sequences derived from samples (mostly planktonic) collected in various freshwater lakes worldwide. Our screening of environmental DNA extractions from a selection of terrestrial and marine habitats using two complementary PCR strategies with distinct clade‐specific primers (see Fig. 1 and Table 1) reveals that although these ‘core’ Aquavolonida (lineage NC10‐A) seem to represent the bulk of the diversity of the clade, many more phylotypes basal to them can be found in other terrestrial habitats, from soils to freshwater sediments. To date, however, members of Aquavolonida remain undetected in marine environments, even in large‐scale, broadly targeted surveys such as BioMarKs (Logares et al. 2014) and *Tara*‐Oceans (de Vargas et al. 2015), which readily detect NC10 when applied to freshwater samples (Lara et al. 2015; Grossmann et al. 2016; Yi et al. 2017).

The results of our environmental DNA screening also provide information about the habitat range and relative diversity of each Aquavolonida lineage. First, both PCR strategies show that NC10‐A are not exclusively planktonic, but can also be found in freshwater sediments and associated with biofilms. However, PCR strategy I (targeting NC10‐A only) generated significantly more positives (and uncovered a much higher diversity of 18S types) from planktonic samples than from sediments and river biofilm, and most of that diversity belongs to the subclade targeted by our TSA‐FISH probes (NC10‐Ai). Even recently flooded soil/grassland sites produced a high frequency of positive PCR results. Therefore, although our TSA‐FISH results indicate that NC10‐A cells do not necessarily occur in high density in planktonic samples, they appear to be genetically diverse, very widely distributed, and present in most freshwater habitats (predominantly in the water column as genuinely planktonic protists).

By contrast, we could not detect other Aquavolonida lineages in planktonic samples, suggesting that only NC10‐A have adapted to a planktonic lifestyle (see the results obtained with PCR strategy II in Table 1). The most basal lineage we detected (NC10‐D) seems restricted to soils, and was detected at relatively high frequency in such samples. The lineage including the new genus *Aquavolon* (NC10‐B) was found in soils, freshwater sediments, and river biofilm. However, in soils it was detected at lower frequency than NC10‐D, and in freshwater sediments and river biofilm at lower frequency than NC10‐A. NC10‐B and NC10‐D also both seem to comprise a much lower diversity of 18S types than NC10‐A (see Fig. 1).

By providing for the first time phenotypic data on members of Aquavolonida, we show that it contains both swimming (new genus *Aquavolon*) and gliding (isolate Lapot, which will be formally described elsewhere) biflagellates with morphological affinities to *Tremula longifila*, the first described taxon from NC11 (Howe et al. 2011). At present, phenotypic data are available only for two (B & C) of four Aquavolonida lineages. However, (i) their morphological similarity to the more distantly related *Tremula*, (ii) the specific locomotion of *Aquavolon*, (iii) the inference from environmental sequencing that the most diverse, later‐diverging NC10‐A are predominantly found in planktonic habitats and probably absent from soils, (iv) TSA‐FISH results concordant with morphology of *Aquavolon* as shown by light microscopy (Fig. 2), and (v) the strongly supported sister relationship between NC10‐A and NC10‐B, together suggest that these two lineages at least probably comprise more or less similar, eukaryovorous swimming flagellates. As NC10‐A sequences are frequently detected in freshwater plankton environmental sequencing surveys, our findings will enable more informed interpretation of such studies.

Unlike Aquavolonida and Tremulida, previous environmental sequencing results show that members of NC12 are found in a broader range of both marine and nonmarine habitat types including soils, sediments, and water column, which suggests a wider diversity of cell types and perhaps lifestyles. It is interesting that so little of the large diversity in the broader clade comprising NCs 10 to 12 has been isolated into culture. This is perhaps because they have a general requirement for eukaryotic prey, which are relatively infrequently used in isolation attempts. Single cell sorting surveys of freshwater habitats should be more informative about their true abundance, which based on detection frequency representing rich lineage diversity, appears to be high.

Phylogenetically, *Aquavolon* can be most directly compared morphologically with *Tremula*, up to now the only identified protist within NCs 10 to 12 of known phenotype. *Tremula longifila* is similarly a nonamoeboid biflagellate heterotrophic protist with a rounded or elongated or spindle‐shaped cell and, like *Aquavolon,* has a variably pronounced medium notch (Howe et al. 2011). In contrast to *Aquavolon,* the flagella of *T. longifila* do not appear to insert close together at the anterior end of the cell; however, although unconfirmed by ultrastructural studies, the proximal part of the posterior axoneme is likely intracellular (Howe et al. 2011). *Aquavolon* can be distinguished from Cercomonadida, Glissomonadida, and Granofilosea (*Massisteria*) by the absence of a microbody (or paranuclear body) with amorphous contents, which usually joins the posterior part of the nucleus.

*Tremula* is so far unique in Rhizaria with respect to its gliding on both exceptionally long flagella, which are simultaneously in contact with the substratum (Howe et al. 2011). All known metromonads, glissomonads, pansomonads, cercomonads, thecofiloseans, and imbricateans glide on their posterior flagellum only. Very unusually for rhizarian flagellates, but similar to the putative glissomonad *Katabia gromovi* (Karpov et al. 2003), *Aquavolon* swims almost perpetually, members of both taxa rotating along their longitudinal axes. They also share subapical insertion of flagella, the same kinetosome orientations, narrow posterior cell end, and the shape of their mitochondria cristae, but *Aquavolon* lacks microbodies, mushroom‐like bodies (refractile granules), kinetocysts, and cysts.

Eukaryovory is also relatively rare in known rhizarian flagellates. Other examples include the metromonads *Metromonas simplex* and *Metopion fluens*, and the pansomonad *Aurigomonas solis* (Vickerman et al. 2005), which capture whole prey cells, usually bodonids, chrysomonads, or euglenids. *Aquavolon* differs ultrastructurally from metromonads and *Aurigamonas* in lacking large extrusive organelles (or haptosomes: *Aurigamonas*), lacking flagellar hairs, and in having a simple plasma membrane rather than a complex envelope of a thin bilayer membrane and an outer layer of thin short fibers (metromonads) or axopodia‐like appendages (haptopodia) (*Aurigamonas*). Interestingly, both *Aquavolon* and *Aurigamonas* have long kinetosomes, but *Aquavolon* also possesses two wide microtubular bands.

Our Bayesian phylogenetic analysis places NC10 (including *Aquavolon*), Tremulida (NC11), and NC12 as a sister clade to Endomyxa, which includes many amoeboid organisms producing filopodia, rhizopodia, and reticulopodia. Many are obligate endoparasites of land plants, stramenopile algae (e.g. Neuhauser et al. 2014), and animals (e.g. Hartikainen et al. 2014a,b; Ward et al. 2016, 2018), but others are free‐living protists, e.g. the reticulopodial amoeba *Filoreta marina* (Bass et al. 2009a), which also has mitochondria with tubular cristae (Myl'nikov and Myl'nikov 2011). Flagellate cells are apparently relatively rare in Endomyxa. *Gromia* produces flagellated dispersal cells or gametes (Hedley and Bertaud 1962), and plasmodiophorids (Phytomyxea) and paradinids (Ascetosporea) form flagellated zoospores. Other ascetosporeans produce nonflagellated spores (e.g. haplosporidians). *Aquavolon* shares some morphological similarity with the biflagellate zoospores of Plasmodiophorida, in which the kinetosomes are also usually very long: 1.6–1.8 μm in *Polymyxa betae* (Barr and Allan 1982) and *Sorosphaera veronicae* (Talley et al. 1978). However, the kinetosomes of the closely related *Plasmodiophora brassicae* are appreciably shorter (0.5 μm) (Buczacki and Clay 1984). Interestingly, the angle between two kinetosomes in different plasmodiophorids ranges from 150° in *Polymyxa graminis* to 30° in *Woronina pythii* and *Plasmodiophora brassicae* (Barr and Allan 1982; Miller and Dylewski 1983; Buczacki and Clay 1984). The zoospores of *Spongospora subterranea* show a lateral insertion of the flagella at an angle of about 180° to each other and a bulging ring at the points of insertion (Merz 1992).

Recent phylogenomic analyses focusing on Rhizaria show a basal split between two major assemblages, Cercozoa (=Filosa) on the one side, and Endomyxa plus Retaria on the other side (e.g. Sierra et al. 2016; Krabberød et al. 2017). In this context, the phylogenetic position of the clade comprising Aquavolonida, Tremulida, and NC12 (if they are confirmed to form a clade) becomes of particular significance. 18S rRNA gene phylogenies place this clade between these two major assemblages of Rhizaria, which makes it a potential third lineage at the deepest rhizarian divergence. On the basis of a shared 18S rRNA sequence signature (Table 2), we hypothesize that the Aquavolonida–Tremulida–NC12 clade may be sister to the highly diverse radiation of Endomyxa + Retaria, and that the ancestral rhizarian phenotype was likely a unicellular heterotrophic biflagellate. Phylogenomic and comparative genomic analyses including members of Aquavolonida and Tremulida are now required to resolve the deepest relationships within Rhizaria.

## Taxonomic summary

We describe the new order Aquavolonida to include all members of NC10 on the basis of its phylogenetic (high statistical support, unifying sequence signatures) and ecological (seemingly exclusively nonmarine) coherence. This name, based on the only described genus, reflects the tendency of the clade to adapt to an aquatic habitat, but is not intended to imply that all lineages are actual swimmers.

### Eukaryota; Rhizaria; Aquavolonida ord. nov. Bass and Berney

#### Diagnosis

Defined as the least inclusive clade containing the last common ancestor of the rhizarian lineages sharing a unique combination of 18S rRNA sequence signatures consisting of two complementary substitutions from U‐A to G‐C and from G‐C to C(U)‐G(A) in helix 11, one complementary substitution from A‐T to C(T)‐G(A) in helix 48, and a specific motif of two adjacent substitutions (AU instead of YG) in a nonbinding part of the 3′ stem of helix 25 (all bold and underlined in Table 2), and all their descendants.

#### Remarks

This is a node‐based definition intended to apply to a crown clade; qualifying clause: the name does not apply if any of the following fall within the specified clade: *Tremula longifila* Howe and Cavalier‐Smith 2011, or the organisms matching environmental clones MPE2‐26, CCW‐46, NAMAKO‐20, and CCA33. Aquavolonida corresponds to the rhizarian clade previously referred to as Novel Clade 10 as identified in this study, i.e. consisting of at least four lineages, one of which contains *Aquavolon* n. gen. The sequence signatures defining the order are found in these four lineages, but we cannot exclude that they were secondarily lost or reversed in as yet unidentified members of the order with a more divergent 18S rRNA sequence. Based on existing evidence, other members of the order will likely but not necessarily all be biflagellated, eukaryovorous, unicellular protists, and are expected to be restricted to terrestrial habitats. Lineages within Aquavolonida are here hypothesized to show a gradual morphological and behavioral adaptation to an aquatic lifestyle with a swimming locomotion, hence, the name chosen for the order.

#### Etymology

Derived from *Aquavolon*, first described genus in the order, and likely to represent its most common phenotype.

## Aquavolon n. gen. Tikhonenkov, Mylnikov, and Bass

### Diagnosis

Unicellular protist with two smooth subapical heterodynamic flagella. The flagella kinetosomes lie approximately at a right angle to each other and are connected by at least one fibril. The posterior kinetosome is very long (> 1 μm). Cell slightly metabolic, not flattened, with remarkable lateral depression in the middle lateral point of the cell body. Single contractile vacuole and nucleus located anteriorly. Several mitochondria have tubular cristae. Rapidly swimming and rarely gliding protist. Eukaryovorous.

### Etymology

From aqua = water, Latin, and volo = to fly, Latin.

### Zoobank Registration

urn:lsid:zoobank.org:act:635F34CC‐265E‐4F6C‐88A4‐C0EC7E84E778

### Type species

*Aquavolon hoantrani*.

## *Aquavolon hoantrani* n. sp. Tikhonenkov, Mylnikov, and Bass

### **Diagnosis**

Cells are elongated oval with lateral depression, 7‐10 μm long, and 3.5–5 μm wide. The anterior end is wider than posterior one and both are roundish. The anterior flagellum is 1.5–2 times longer than its cell body length, makes flapping movements, and directs forward at cell swimming. The posterior flagellum is about 2–3 times the cell length, lies along the cell body at the anterior part of the cell, and stands apart from the cell at the level of lateral depression, directs backward, and trails behind the cell. Reproduction or resting cysts have not been found in culture.

### Type material

A block of chemically fixed resin‐embedded cells of the type strain, Colp‐16, is deposited in Marine Invertebrate Collection, Beaty Biodiversity Museum, University of British Columbia as MI‐PR210. This constitutes the name‐bearing type of the new species (a hapantotype).

### Type Figure

Figure 2A illustrates a live cell of strain Colp‐16.

### Type locality

Water with bottom detritus from freshwater Suoi Da Reservoir (Hồ Suối Đá, viet.), Bình Thuận Province, S.R. Vietnam.

### Etymology

Named after Dr. Hoan Tran, Vietnamese biologist and ecologist, who significantly contributed to sample collection and field trips organization and management in S.R. Vietnam.

### Gene sequence

The 18S rRNA gene sequence has the GenBank Accession Number MG760572.

### Zoobank Registration

urn:lsid:zoobank.org:act:3E585A8A‐9459‐4E1F‐956B‐FE547535EE1F

## *Aquavolon dientrani* n. sp. Tikhonenkov, Mylnikov, and Bass

### Diagnosis

Elongated‐oval cells 8.5–11 μm long with wider anterior end and lateral depression. Anterior flagellum is about the half of cell length or slightly longer, makes fast flapping movements at swimming, and often invisible, may wraps around the anterior part of the cell. The posterior flagellum is 2–2.5 times longer than the cell body, stands apart from the cell at the level of lateral depression, and trails behind the cell. Cells can form short, relatively wide pseudopodia posteriorly or produce single tail pseudopodium. Reproduction or resting cysts have not been found in culture.

### Type Figure

Figure 2I illustrates a live cell of *A. dientrani*.

### Type locality

Wetland water with decaying plant material, Đắc Lua Village, Dong Nai Province, S.R. Vietnam.

### Etymology

Named after Mr. Tran Duc Dien, Vietnamese biologist and ecologist, who significantly contributed to sample collection and field trips organization and management in S.R. Vietnam.

### Gene sequence

The 18S rRNA gene sequence has the GenBank Accession Number MG818163.

### Zoobank Registration

urn:lsid:zoobank.org:act:C99A3C2D‐AF23‐4A48‐ACE0‐377340713E8C

## Supporting information


**Table S1.** Aquavolonida sequences in GenBank.
**Table S2.** Sequences of the PCR primers used in this study.
**Video S1.** *Aquavolon dientrani* swimming. Video can be accessed at https://yadi.sk/mail/?hash=jsf7XwEScPTOsgvjNRSf5UTcbJ9iAjh6mG6I4cGmALw%3D
