# Supplementary material for: Rhizarian ‘Novel Clade 10’ Revealed as Abundant and Diverse Planktonic and Terrestrial Flagellates, including Aquavolon n. gen
Source: J Eukaryot Microbiol. 2018 May 14;65(6):828–42. doi: 10.1111/jeu.12524 (PMC6282753; doi:10.1111/jeu.12524)
Supplement: Supplementary file 1 — Table S1. Aquavolonida sequences in GenBank. Table S2. Sequences of the PCR primers used in this study. Video S1. Aquavolon dientrani swimming. Video can be accessed at https://yadi.sk/mail/?hash=jsf7XwEScPTOsgvjNRSf5UTcbJ9iAjh6mG6I4cGmALw%3D [file JEU-65-828-s001.pdf]

SUPPORTING INFORMATION

Rhizarian 'Novel Clade 10' Revealed as Abundant and Diverse Planktonic and Terrestrial Flagellates, including *Aquavolon* n. gen.by David Bass, Tikhonenkov DV, Foster R, Dyal P, Janouskovec J, Keeling PJ, Gardner M, Neuhauser S, Hartikainen H, Mylnikov AP, Berney C

Table S1. Aquavolonida sequences in GenBank.

Table S2. Sequences of the PCR primers used in this study.

Video S1. *Aquavolon dientrani* swimming. Video can be accessed at https://yadi.sk/mail/?hash=jsf7XwEScPTOsgvjNRSf5UTcbJ9iAjh6mG6I4cGmALw%3D

Supplementary Table 1. Aquavolonida sequences in GenBank.

Accession No	Clone	Length	Lineage	Sample locality and type	Reference
AY919680	LG01-12	1729	NC10-A1	Lake George, North-Eastern New York, USA: euphotic water column (oligotrophic) - summer 1996	Richards et al. (2005) Env. Microbiol. 7:1413-1425.
AY919754	LG21-01	1737	NC10-A1		
AY642739	A50	1298	NC10-A1	Lake Aydat, Massif Central, France: euphotic water column (eutrophic) - Aug 2002	Lefranc et al. (2005) Appl. Environ. Microbiol. 71:59
DQ243999	PCB7AU2004	1803	NC10-A3	Lake Pavin, Massif Central, France: oxycline (suboxic) water column (oligomesotrophic) - Aug 2004	Lefèvre et al. (2007) Env. Microbiol. 9:61-71.
EF196690	BA74	834	NC10-A3	Lake Bourget, Alps, France: water column (mesotrophic) - May/Aug 2005	Lepère et al. (2008) Appl. Environ. Microbiol. 74:294
EF196697	BA272	814	NC10-A3		
EF196707	BA400	810	NC10-A3		
EF196716	B9	879	NC10-A2		
EF196754	BI105	807	NC10-A2		
EF196757	BI110	900	NC10-A1		
EF196759	BI113	821	NC10-A2		
EU143892	MLB90.167	800	NC10-A2	Meiliang Bay, Lake Taihu, China: water column (eutrophic, shallow, subtropical) - Nov 2006	Chen et al. (2008) Microb. Ecol. 56:572-583.
EU162632	PCC12SP2005	1796	NC10-A1	Lake Pavin, Massif Central, France: oxic water column (oligomesotrophic) - May/Jun/Jul 2005	Lefèvre et al. (2008) PLoS ONE 3:e2324.
EU162633	PCA3SP2005	1794	NC10-A1		
FJ349632	051011_T2S1_W_T_	1065	NC10-A2	Lake Pontchartrain, Louisiana, USA: water column - Oct 2005	Amaral-Zettler et al. (2008) Environ. Sci. Technol. 4
FJ353183	C7p3_ML_153	1066	NC10-A2		
FJ410720	EBM54.96	800	NC10-A2	East Bay, Lake Taihu, China: water column (oligotrophic, shallow, subtropical) - date unspecified	Chen (2008) unpublished
FJ410763	EBA63.28	800	NC10-A2		
EU567283	DB-2703-3	1121	NC10-A1	unspecified: shallow anaerobic sediment - date unspecified	Bass et al. (2009) Protist 160:75-109.
EU567284	DB-2703-4	1053	NC10-A2		
GU067900	ESS270706.007	793	NC10-A2	Lake Esch-sur-Sure, Luxembourg: water column (meso-eutrophic) - Jul 2006	Masquelier et al. (2010) unpublished
GU067902	ESS270706.009	777	NC10-A2		
GU067903	ESS270706.010	845	NC10-A2		
GU067911	ESS270706.018	791	NC10-A2		
GU067921	ESS270706.028	1795	NC10-A2		
GU067929	ESS270706.036	866	NC10-A2		
GU067956	ESS270706.063	867	NC10-A2		
GU067966	ESS270706.073	790	NC10-A2		
GU067974	ESS270706.081	784	NC10-A2		
GU067979	ESS270706.086	830	NC10-A2		
GU067980	ESS270706.087	788	NC10-A2		
GU067986	ESS270706.093	835	NC10-A2		
HQ219347	AY2009C14	2567 (1775)	NC10-A3	Lake Aydat, Massif Central, France: euphotic water column (eutrophic) - Jul 2008	Monchy et al. (2011) Env. Microbiol. 13:1433-1453.
AB622281	K4JUN2009	1672	NC10-A1	Lake Kusaki, Gunma, Japan: water column - Jun/Jul/Aug 2009 - May 2010	Fujimoto et al. (2011) unpublished
AB622282	K5JUN2009	1686	NC10-A2		
AB622285	K1JUL2009	1707	NC10-A2		
AB622286	K2JUL2009	1686	NC10-A2		
AB622288	K4JUL2009	1694	NC10-A1		
AB622289	K5JUL2009	1688	NC10-A1		
AB622294	K1AUG2009	1672	NC10-A1		
AB622301	K8AUG2009	1708	NC10-A2		
AB622334	K3MAY2010	1716	NC10-A2		
AB622335	K4MAY2010	1686	NC10-A1		
JN207857	FP989	865	NC10-A2	Fresh Pond, McMurdo Ice Shelf, Antarctica: freshwater microbial mat - Jan 2005	Jungblut et al. (2012) FEMS Microbiol. Ecol. 82:416
HE655134	PC09.H3_BGr_2	931	NC10-A1	Lake Bassa Granotes, Central Pyrenees, Spain: lake plankton - Jul 2008	Triadó-Margarit & Casamayor (2012) Env. Microbiol
HE655237	PC09.B8_Lle_2	807	NC10-A3	Lake Llebreta, Central Pyrenees, Spain: lake plankton - Jul 2008	
HE655261	PC10.D9_Lle_5	808	NC10-A3		
HE655270	PC10.G11_Lle_1	929	NC10-A3		
HE655275	PC10.H1_Lle_1	806	NC10-A3		
HE655276	PC10.H10_Lle_2	794	NC10-A3		
HE655342	PC11.B5_MdA_4	810	NC10-A3	Lake Muntanyó d'Àrreu, Central Pyrenees, Spain: lake plankton - Jul 2008	
HE655347	PC11.F2_MdA_1	809	NC10-A3		
HE655359	PC04.D3_Pla_1	933	NC10-A1	Lake Plan, Central Pyrenees, Spain: lake plankton - Jul 2008	
JF730776	Ch8A2mE7	1705	NC10-A2	Char Lake, Canada: Arctic lake water column - Aug 2008	Charvet et al. (2012) Polar Biol. 35:733-748.
AB721017	DW4_2010	1677	NC10-A1	Kiryu, Gunma, Japan: Motojuku water purification plant - 2010/2011	Fujimoto et al. (2012) unpublished
AB721019	DW6_2010	1691	NC10-A2		
AB721026	RW2_2010	1682	NC10-A1		
AB721028	RW4_2010	1684	NC10-A2		
AB721039	RW15_2010	1702	NC10-A2		
AB721040	RW16_2010	1677	NC10-A2		
AB721071	DW2_2011	1700	NC10-A2		
AB721073	RW1_2011	1700	NC10-A2		
AB721075	RW3_2011	1694	NC10-A2		
AB771803	K6JUN2010	1684	NC10-A2	Lake Kusaki, Gunma, Japan: water column - Jun/Aug/Oct/Nov/Dec 2010 - Jan 2012	Fujimoto et al. (2012) unpublished
AB771806	K9JUN2010	1673	NC10-A1		
AB771807	K10JUN2010	1674	NC10-A1		
AB771817	K2AUG2010	1686	NC10-A2		
AB771825	K10AUG2010	1678	NC10-A1		
AB771830	K3OCT2010	1688	NC10-A2		
AB771831	K4OCT2010	1684	NC10-A1		
AB771834	K7OCT2010	1676	NC10-A2		
AB771836	K9OCT2010	1682	NC10-A1		
AB771845	K8NOV2010	1680	NC10-A1		
AB771848	K11NOV2010	1677	NC10-A1		
AB771849	K12NOV2010	1685	NC10-A1		
AB771863	K14DEC2010	1678	NC10-A1		
AB771903	K10JAN2012	1674	NC10-A1		
JN547284	BR2-5	1588	NC10-A1	Lake Baikal, Russia: water column - date unspecified	Lepère et al. (2012) unpublished
KC306544	BT_Euk_C4	1199	NC10-A3	Hainich National Forest, Germany: groundwater from karstified limestone aquifer - Jun 2010	Risse-Buhl et al. (2013) J. Eukaryot. Microbiol. 60:4
AB996630	O5	894	NC10-A1	Lake Biwa, Shiga, Japan: lake hypolimnion - date unspecified	Mukerjee et al. (2014) unpublished

Supplementary Table S2. Sequences of the PCR primers used in this study.

Primer name	Primer sequence	Sense	Position [a]	Specificity	PCR strategy [b]
**V2f-NC**	CGR RGC YAG RTC ATT CAA ATT TCT G	forward	246-270	all NC10	2
**V4f-nc10**	GCA GGC TTA TTG CTG TGA ATA C	forward	749-770	core NC10	F
**C5f-NC**	GGC CAG AGG TGA AAT TCT ATG	forward	871-891	all NC10	2
**C6r-nc10**	GCT CCA CTT CTT GGG TGC	reverse	1129-1147	core NC10	F
**s1259F**	AGG ATT GWC AGR TTG MAG HTC	forward	1201-1221	all Cercozoa	E
**C7f-NC10**	GAT TGA TCT GTC TGG TTA ATT CCG A	forward	1268-1292	core NC10	1
**C7r-nc10**	ATC GGA ATT AAC CAG ACA GAT CAA	reverse	1270-1293	core NC10	F
**V8r-NC**	GGT TRT AWR CTC GCT GAW HGC AAC	reverse	1451-1474	all NC10	2
**V8f-NC10**	GTT GCG ATC ARC GAG YWT ACA ACC T	forward	1451-1475	core NC10	1
**C9r-NC**	CGT ART CAA TGC AYG CTG ATG ACR	reverse	1571-1594	all NC10	2
**EndoR1**	CGA CTT CTC CTT CCT CTA ARY RDT AWG	reverse	1710-1736	anti-Filosa	1 and E
**sB2n**	CCT ACG GHD ACC TTG TTA CGA CTT CTC	reverse	1728-1754	anti-Filosa	1 and E

[a] Position given using NC10 environmental clone AY2009C14 (accession number HQ219347) as a reference.
[b] 1 = primers used to explore core ("planktonic") NC10 diversity; 2 = primers used to explore the whole NC10 diversity; E = primers used to generate Endomyxa-enriched clone libraries; F = primers matching the FISH probes designed to target part of core NC10.
